# A Principal Component of Quality of Life Measures Is Associated with Survival for Head and Neck Cancer Patients Treated with Radiation Therapy

**DOI:** 10.3390/cancers13051155

**Published:** 2021-03-08

**Authors:** Mark Farrugia, Han Yu, Sung Jun Ma, Austin J. Iovoli, Kristopher Attwood, Kimberly E. Wooten, Hassan Arshad, Vishal Gupta, Ryan P. McSpadden, Moni A. Kuriakose, Michael R. Markiewicz, Jon M. Chan, Wesley L. Hicks, Mary E. Platek, Andrew D. Ray, Elizabeth A. Repasky, Anurag K. Singh

**Affiliations:** 1 Department of Radiation Medicine, Roswell Park Comprehensive Cancer Center, 665 Elm Street, Buffalo, NY 14203, USA; Mark.Farrugia@roswellpark.org (M.F.); SungJun.Ma@RoswellPark.org (S.J.M.); Austin.Iovoli@RoswellPark.org (A.J.I.); Mary.Platek@RoswellPark.org (M.E.P.); 2 Jacobs School of Medicine and Biomedical Sciences, University at Buffalo, The State University of New York, 955 Main Street, Buffalo, NY 14203, USA; 3 Department of Biostatistics and Bioinformatics, Roswell Park Comprehensive Cancer Center, 665 Elm Street, Buffalo, NY 14203, USA; Han.Yu@RoswellPark.org (H.Y.); Kristopher.Attwood@RoswellPark.org (K.A.); 4 Department of Head and Neck Surgery, Roswell Park Comprehensive Cancer Center, 665 Elm Street, Buffalo, NY 14203, USA; Kimberly.Wooten@RoswellPark.org (K.E.W.); Hassan.Arshad@RoswellPark.org (H.A.); Vishal.Gupta@RoswellPark.org (V.G.); Ryan.McSpadden@RoswellPark.org (R.P.M.); Moni.Kuriakose@RoswellPark.org (M.A.K.); Michael.Markiewicz@RoswellPark.org (M.R.M.); Jon.Chan@RoswellPark.org (J.M.C.); Wesley.Hicks@RoswellPark.org (W.L.H.J.); 5 Department of Oral and Maxillofacial Surgery, School of Dental Medicine, University at Buffalo, The State University of New York, 3435 Main Street, Buffalo, NY 14214, USA; 6 Department of Neurosurgery, Department of Surgery, Jacobs School of Medicine and Biomedical Sciences, University at Buffalo, The State University of New York, 955 Main Street, Buffalo, NY 14203, USA; 7 Department of Cancer Prevention and Control, Roswell Park Comprehensive Cancer Center, 665 Elm Street, Buffalo, NY 14203, USA; Andrew.Ray@RoswellPark.org; 8 Department of Dietetics, D’Youville College, 270 Porter Avenue, Buffalo, NY 14201, USA; 9 Department of Immunology, Roswell Park Comprehensive Cancer Center, 665 Elm Street, Buffalo, NY 14203, USA; Elizabeth.Repasky@RoswellPark.org

**Keywords:** quality of life, head and neck neoplasms, survival, principal component analysis

## Abstract

**Simple Summary:**

Health-related quality of life (HRQOL) surveys describe the patient experience of disease and treatment. The relationship between post-treatment recovery of HRQOL and outcome in head and neck cancer is not well characterized. Impaired recovery of numerous individual components of HRQOL were associated with increased mortality. To obtain a better understanding how HRQOL (as a whole) impacts survival, we utilized a statistical technique called principal component analysis (PCA). PCA generated a total score of several HRQOL domains, named principal component 1 (PC1), to more accurately describe the cumulative impact of poor HRQOL recovery on outcome. PC1 was associated with survival and may be a useful tool in future studies to identify at-risk patients.

**Abstract:**

Background: Health-related quality of life (HRQOL) metrics can be associated with survival in head and neck cancer (HNC); however, the impact of HRQOL recovery and the relevant HRQOL domains regarding outcome are unclear. Methods: Using a single-institution database, we retrospectively reviewed HNC patients treated with definitive or postoperative radiation therapy between 2013 and 2018. The recovery of individual HRQOL domains were determined by the ratio of the post-treatment to baseline scores. Univariate and Multivariate Cox regression were used to analyze survival outcomes. Principal component analysis was used to adjust for multicollinearity of HRQOL domains. Results: In 218 HNC patients who received radiation therapy, median follow-up was 24.8 months (interquartile range (IQR) 14.5–32.0). Principal component analysis evaluating the recovery of HRQOL domains revealed two independent principal components (PC), PC1 and PC2. PC1, which received contributions from the functional domains; physical (PF), role (RF), emotional (EF), cognitive (CF), and global health status (GQOL) was significantly associated with disease-free (HR = 0.77, 95% CI 0.61–0.98, *p* = 0.034) and overall survival (HR = 0.76, 95% CI 0.65–0.91, *p* = 0.004) on multivariate analysis and PC2, had no correlation with outcome and was mainly represented by social functioning. Unplanned hospitalization was significantly associated with lower PC1 scores (β = −0.997, Std. Error = 0.244, *p* < 0.001). Conclusion: Our study provides evidence that post-treatment recovery of HRQOL domains were associated with overall survival (OS) in HNC. PC1 is an attractive clinical tool to assess the recovery across multiple different HRQOL and the relationship with survival. Future prospective studies may identify patients who could benefit from additional rehabilitation based on PC1 score.

## 1. Introduction

Head and neck cancers (HNC) of the oral cavity, pharynx, and larynx are cumulatively within the top 10 most commonly diagnosed adult cancers worldwide with an estimated 15 new cases and 6.5 deaths per 100,000 people in 2020 [1]. Outcomes can vary significantly among HNC with human papilloma virus (HPV)-associated tumors of the oropharynx approaching a survival rate of 80–90%, yet locally advanced, HPV-negative cancers of the oropharynx and other subsites demonstrate an approximate 5-year survival of 50–60% [2,3,4]. Radiation therapy is considered one of the main treatment modalities for HNC and is utilized in over 60% of patients [5]. Regrettably, treatment for HNC is associated with considerable morbidity with a substantial impact on health-related quality of life (HRQOL) [6]. To better capture the HRQOL effects of treatment, incorporation of health-related quality of life (HRQOL) outcomes is considered standard practice in modern clinical trials [7]. Interestingly, while these metrics were designed to characterize the patient experience of disease and treatment sequalae, a growing body of evidence suggests HRQOL parameters can have prognostic implications [8,9,10,11,12,13,14,15,16,17,18,19].

The European Organisation for Research and Treatment of Cancer (EORTC) Quality of Life Questionnaire C30 (QLQ-C30) is a well-validated questionnaire which provides information across several HRQOL domains [20]. Within HNC, several groups have demonstrated the prognostic utility of these measures; however, the relevant functional domain and the time point as which it is studied are heterogenous across reports [13,14,15,17,18,19,21,22,23,24,25]. For example, reports have shown pre-treatment metrics for physical functioning to yield prognostic importance in HNC [22,25], however, others observed similar findings with baseline emotional function, global quality of life, or sum HRQOL scores [14,15,23,24]. Similarly, when evaluating trends after completion of therapy or pre- and post-treatment comparisons, changes in physical functioning and global quality of life have been both associated with survival [18,19,21]. Other considerations, such as the degree to which post-treatment HRQOL scores return to or exceed baseline metrics, have received little attention [17]. It is unclear whether these discrepancies can be attributed to differences in timepoints, measures employed, or whether certain domains are excluded in multivariate analyses secondary to covariance [26,27].

Given these inconsistencies, practical implementation of HRQOL measures as clinical prognostic factors is limited. Therefore, using an institutional database of HNC patients treated with definitive or adjuvant radiotherapy, we investigated the relationship between post-treatment recovery of HRQOL measures and survival-based outcomes. Furthermore, to potentially improve the reproducibility of these findings, we employed principal component analysis to account for multicollinearity of HRQOL domains.

## 2. Methods

### 2.1. Patient Population

The institutional review board approved this previously characterized retrospective database review of HNC patients treated at Roswell Park Comprehensive Cancer Center (RPCCC; Buffalo, NY, USA) who received definitive or post-operative radiotherapy (RT) [28]. Within this group, 303 had completed baseline EORTC-QLQ-C30 questionnaires ranging from 2013–2018. Of these patients, 218 also had also completed a post-treatment survey. This analysis was performed with a waiver for consent under approval by the Roswell Park Comprehensive Cancer Center Institutional Review Board for human subject protection (EDR-103707).

### 2.2. Clinical Data

Demographic data were recorded to account for potential confounding factors between HRQOL and outcome. Variables of interest included age, gender, performance status, head and neck cancer subsite, and receipt of chemotherapy. Missing data comprised no more than 1% for any variable. Patients were considered former smokers if they had successfully stopped smoking within 30 days of starting treatment [29]. Staging was completed by the American Joint Committee on Cancer 7th edition. Radiation dose and volumes have been previously characterized [30,31]. Squamous cell carcinoma (SCC) was the histology in over 98% of the cases. Regarding the salivary gland subsite, 4 of the 5 tumors were SCC involving an intraparotid gland lymph node. Human papilloma virus (HPV) status was determined by p16 positivity via immunohistochemistry and only routinely tested for cancers involving the pharynx. Histologic grade is not routinely reported at our institution for all HNC cases and in the absence of this information, grade was defined as unknown. Unplanned hospitalization was defined as previously described [28]. Nutritional support was defined as the use of a feeding tube or total parenteral nutrition. Relapse was defined either by radiographic progression or biopsy. Relapse-free survival (RFS), disease-specific survival (DSS), and overall survival (OS) were calculated using date of 1st treatment to date of the respective event. For RFS, relapse was termed an event, and patients who did not relapse or were deceased without a history of relapse were censored. For DSS, patients who died with a history of relapse were termed an event, all others were censored. Regarding OS, death due to any cause was termed an event and all others were censored.

### 2.3. Quality of Life Questionnaires

HRQOL was assessed via the EORTC QLQ-C30 [20]. EORTC QLQ-C30 is comprised of 30 items incorporating five functional scales (physical (PF), role (RF), social (SF), cognitive (CF), emotional (EF)) and a global health status (GQOL) (Supplementary Materials). Raw metrics were converted to a 0–100 score, where a greater number denotes higher function. Baseline questionnaires were completed prior to or within 7 days of the start of treatment. Post-treatment surveys were typically recorded on follow-up 3 months after completion of RT. To characterize recovery, we assessed the ratio of post-treatment to baseline scores within each HRQOL domain, subsequently categorizing this metric on an ordinal scale: equal to or exceeded (Complete recovery) = 0, (more than 80%) = 1, and (<80%) = 2. For example, if a patient’s post-treatment and baseline PF score were 70 and 100, respectively, they would be scored as 2, representing <80% for PF recovery.

### 2.4. Statistical Analysis

Demographic groups were assessed using the Pearson Chi-square test for categorical variables and the Wilcoxon test for continuous variables. Univariate and multivariate (MVA) Cox regression, along with Kaplan–Meier survival estimation were used to examine relationships with RFS and OS. To select variables for MVA, variables were included if *p*-values were <0.1 on univariate study. Similarly, linear regression was used to identify correlative variables with PC1 and those with *p*-values of <0.1 on univariate study were included in the multivariate model.

Principal component analysis (PCA) was used for dimension reduction, which linearly transformed the five functional scales and global status of HRQOL into orthogonal components, where the variances of the leading components were maximized. Intuitively, the PCA was used to reduce the number of variables while preserving as much information in the original data as possible. As above, recovery was coded such that Complete recovery = 0, more than 80% = 1, and <80% = 2. Projection of original HRQOL variables as well as the data points onto the first two PCs were visualized using the biplot. In this study, we focused on the first principal component as other components were found to be uncorrelated with the outcomes of interest. Specifically, the PC1 can be calculated as where each variable was normalized as Z-scores before calculating PC1 (formula seen below). Coefficients for all principal components can be found in Supplementary Materials.

*PC*1 = 0.125*SF* − 0.398*CF* − 0.363*EF* − 0.475*RF* − 0.505*PF* − 0.461*GQOL* (1)

All *p*-values were two-sided. Variables with *p* ≤ 0.05 were considered significant. Statistical analyses were performed using Graph Pad Prism Version 8.4.3, IBM SPSS Version 26, and R version 4.0.2.

## 3. Results

A total of 218 patients who fulfilled our inclusion criteria completed pre- and post-treatment HRQOL surveys. The median age was 61.4 years (interquartile range (IQR) 54.6–67.2 years); median follow-up was 24.8 (IQR 14.5–32.0) months; the majority of the group was male (*n* = 173, 79.4%), Karnofsky performance status (KPS) 90–100 (*n* = 164, 75.3%), and former smokers (*n* = 142, 65.4%) (Table 1). Tumor histology was squamous cell carcinoma in more than 98% of the cases. Of all patients, 126 (57.8%) had pharyngeal tumors, 89 (70.6%) of which were associated with HPV. Tumor stage was most commonly T2 (*n* = 63, 29.0%) and T3 (*n* = 65, 30.0%); N2 (*n* = 126, 58.3%) was the most common nodal stage. A small number of patients (*n* = 12, 5.5%) underwent induction chemotherapy. Most underwent concurrent chemoradiation (*n* = 196, 89.6%). Adjuvant radiation was given to 51 patients (23.4%). Unplanned hospitalization occurred in 39 (22.3%) patients either during or within 90 days of completing treatment. Within the follow-up period, 48 (22.0%) patients had had a relapse of the disease and 42 (19.3%) patients had died.

Pre-treatment and post-treatment scores for the HRQOL domains and global health status and distribution of recovery are shown in Table 2. At three months post treatment, HRQOL scores were significantly different for the PF (*p* = 0.0001) and RF domains (*p* = 0.001). With regard to HRQOL recovery, the highest proportion of patients with scores <80% of baseline was observed in RF (*n* = 60, 27.5%), whereas fewer patients demonstrated residual deficits in CF (more than 80%: *n* = 31, 14.2%, <80%: *n* = 18, 8.3%). Mean and standard deviation for the recovery of each HRQOL domain are shown in Table 2.

Univariate Cox regression analysis results are shown in Table 3. No recorded variables were significantly associated with RFS; however, statistical trends were observed with pharynx primary (Hazard’s ratio (HR) 0.43, 95% confidence interval (CI) 0.18–1.0, *p* = 0.061), N3 disease (HR = 2.55, 95% CI 0.95–6.8, *p* = 0.062), former smokers (HR = 0.48, 95% CI 0.20–1.1, *p* = 0.097), nutritional support (HR = 1.7, 95% CI 0.93–2.9, *p* = 0.09), and impaired recovery of RF (HR = 1.3, 95% CI 0.96–1.8, *p* = 0.095), and CF (HR = 1.4, 95% CI 0.94–2.1, *p* = 0.097).

Regarding DSS, pharynx primary (HR = 0.24, 95% CI 0.08–0.75, *p* = 0.014), T1–T2 tumor stage (*p* < 0.05), HPV positivity (HR = 0.37, 95% CI 0.15–0.92, *p* = 0.032), never smokers (HR = 0.13, 0.03–0.66, *p* = 0.013), nutritional support (HR = 2.6, 1.1–6.0, *p* = 0.028), and impaired recovery of GQOL (HR = 1.8, 95% CI 1.1–2.9, *p* = 0.015) were significantly associated factors.

Several variables were significantly associated with OS, including KPS (HR = 0.88, 95% CI 0.59–0.89, *p* = 0.006), pharynx primary (HR = 0.29, 95% CI 0.08–0.72, *p* = 0.008), salivary gland subsite (HR = 0.19, 95% CI 0.04–0.93, *p* = 0.040), T0–T3 tumor stage (*p* > 0.03), HPV positivity (HR = 0.34, 95% CI 0.16–0.7, *p* = 0.004), never smokers (HR = 0.26, 95% CI 0.09–0.76, *p* = 0.014), nutritional support (HR = 2.6, 95% CI 1.4–5.1, *p* = 0.004), and recovery of RF (HR = 1.6, 95% CI 1.1–2.2, *p* = 0.009), EF (HR = 1.8, 95% CI 1.3–2.6, *p* = 0.001), and GQOL (HR = 1.3, 95% CI 1.2–2.4, *p* = 0.006).

**Figure 1:**
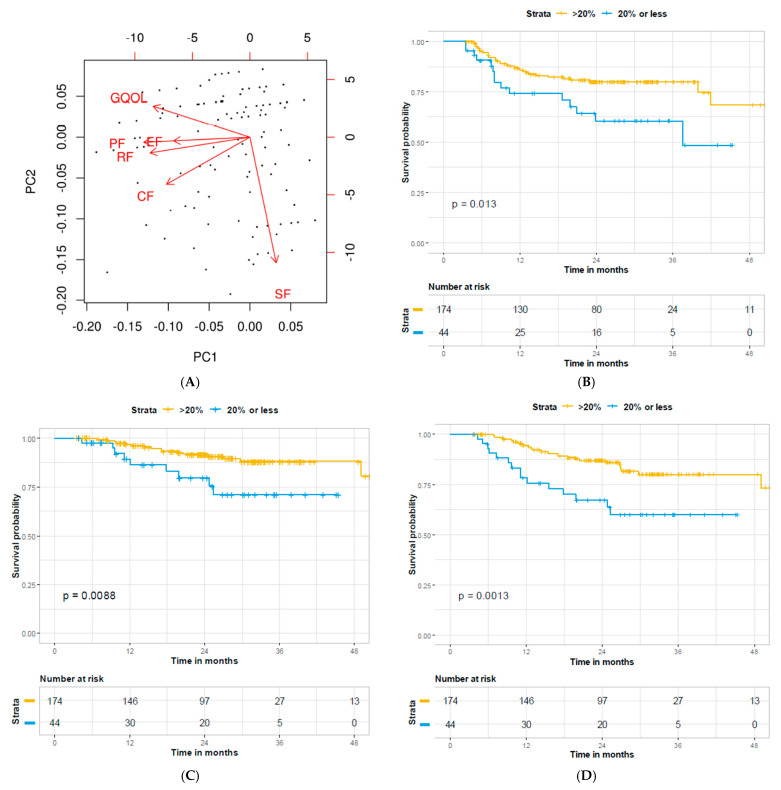
Principal component analysis of HRQOL recovery. (**A**) The biplot shows the projection of HRQOL scales on PC1 and PC2, as well as the sample distribution of the data set. RFS (panel **B**), DSS (panel **C**), and OS (panel **D**) stratified by the bottom 20% of PC1 scores.

To adjust for multicollinearity of HRQOL domains, PCA was performed. We examined the two principal components (PC) 1 and 2 (Figure 1A), which explain 40% and 17% of the total variance respectively. Coefficients for all principal components can be found in supplementary materials. The biplot demonstrates that PF, RF, CF, EF, and GQOL are positively correlated and contribute primarily to PC1, whereas SF is independent of the other measures and mainly contributes to PC2. This suggests that PC1 can be considered as a weighted summary score for the corresponding scales. Based on the biplot, the projections of HRQOL scales point towards the negative direction of PC1, therefore, a higher PC1 score corresponds to a better recovery. Univariate Cox regression was performed to assess the association between PC1/2 and outcome (Table 3), demonstrating RFS (HR = 0.83, 95% CI 0.70–0.99, *p* = 0.039), DSS (HR = 0.74, 95% CI 0.59–0.94, *p* = 0.013), and OS (HR = 0.74, 95% CI 0.62–0.89, *p* = 0.001) to significantly correlate with PC1 but not PC2 (RFS (HR = 1.2, 95% CI 0.87–1.6, *p* = 0.301), DSS (HR = 1.4, 95% CI 0.88–2.3, *p* = 0.148), OS (HR = 0.98, 95% CI 0.73–1.3, *p* = 0.890)). Examination of the lowest quintile of PC1 scores (scores ≤ −1.28) demonstrates significantly worse RFS (*p* = 0.013), DSS (*p* = 0.009), and OS (*p* = 0.0013) via Kaplan–Meier survival analysis (Figure 1B–D).

Multivariate Cox regression analysis (Table 4) found N3 disease (HR = 0.1, 95% CI 1.4–11.8, *p* = 0.010) to be significantly correlated with RFS; pharynx primary (HR = 0.24, 95% CI 0.08–0.75, *p* = 0.014) and PC1 (HR = 0.77, 95% CI 0.61–0.98, *p* = 0.036) to be significantly associated with DSS; PC1 (HR = 0.76, 95% CI 0.63–0.91, *p* = 0.004) to be significantly associated with OS. By comparison, substituting PF, RF, EF, CF, and GQOL for PC1 into the multivariate model demonstrated none of the above HRQOL domains to be associated with RFS, DSS, or OS. Both univariate and multivariable analyses suggest higher PC1 score correspond to better OS, which implies a better recovery in corresponding scales (PF, RF, CF, EF, and GQOL) is associated with lower risk of death.

Linear regression was used to evaluate whether any factors were associated with PC1 score. In a univariate model, tumor stage (T2 (*p* = 0.02) and T4 (*p* = 0.015)), N3 nodal stage (*p* = 0.08), non-steroidal anti-inflammatory drug use (*p* = 0.076), nutritional support (*p* = 0.006), and unplanned hospitalization (*p* < 0.001) were significantly associated or trended towards association with PC1 (Table 5). Interestingly, KPS was not related to PC1 (*p* = 0.569). On multivariate analysis, unplanned hospitalization (β = −0.997, Std. Error = 0.244, *p* < 0.001) and N3 disease (β = 1.07, Std. Error = 0.429, *p* = 0.013) were significantly associated with PC1 (Table 5).

## 4. Discussion

In the current study, post-treatment HRQOL scores were significantly different from baseline for the PF and RF domains. Approximately 10–25% of patients demonstrated <80% recovery depending on HRQOL domain with recovery of RF, EF, and GQOL correlating with OS on univariate Cox regression. Using PCA, PCs 1 and 2 were identified with an improvement in PF, RF, CF, EF, and GQOL positively correlated with PC1 and SF mainly contributing to PC2. In both univariate and multivariate models, PC1 but not PC2 correlated with DSS and OS. Patients within the lowest quintile of PC1 scores had significantly worse RFS, DSS, and OS. Lastly, factors including unplanned hospitalization and N3 disease were significantly associated with PC1 on linear regression.

Prior reports have demonstrated prognostic utility of different HRQOL metrics in HNC [14,15,18,19,21,22,23,24,25]. Several studies have shown baseline scores of either PF, EF, GQOL, or sum HRQOL metrics to be associated with mortality [14,15,22,23,24,25]. In the post-treatment setting, others have shown PF and GQOL scores or a decline in these metrics to correlate with survival [18,19,21]. By comparison, recovery of HRQOL and how it corresponds to outcome has been less characterized.

Others have previously investigated how improvement of post-treatment functional impairment impacted survival in HNC [17]. Using the Performance Status Scale, which characterizes functional performance based on three scales (Normalcy of Diet, Eating in Public, and Understandability of Speech), authors demonstrated that sustained impairment 3–6 months following treatment of any scale was associated with increased mortality, while those who had recovered from post-treatment deficits did not suffer a worse prognosis. These findings highlighted that treatment-related decline of HRQOL by itself may be incomplete and post-treatment recovery may best identify at-risk patients. In agreement with these observations, in the current study, lower PC1 score which corresponds to worse recovery of HRQOL domains was associated with poor OS [17].

Previous studies have shown certain HRQOL domains to be more informative than others for survival in HNC [15,19,21]. For example, a prior study found baseline PF score and change in PF post treatment to be the strongest predictor for outcome for the HRQOL domains [19]. Others have similarly focused on distinct HRQOL domains typically following multivariate Cox regression demonstrating the superiority of certain domains over others [15,19,21]. One limitation to this approach is that it does not account for the multicollinearity of HRQOL domains. Several studies have demonstrated the multicollinearity of the EORTC-QLQ-C30 domains [26,27,32]. Consistent with these studies, PF, RF, EF, CF, and GQOL were positively correlated in the current investigation. Cox regression, which attempts to control for covariance of variables, may discard certain domains which have a base degree of collinearity. PCA is an alternative approach which can account for multicollinearity and express these covariate factors as independent principal components [33]. In the current study, PCA was used to identify two PCs among the HRQOL domains. PC1 was primarily comprised of PF, RF, EF, CF, GQOL and was significantly associated with OS. PC2 had no correlation with outcome and was mainly represented by SF. In contrast, no individual HRQOL domain was significantly correlated with OS via conventional multivariate Cox regression analysis and this may inappropriately suggest that these domains are dispensable regarding prognosis. PCA may allow for a more accurate characterization between survival and recovery of the HRQOL domains. Additionally, patients within the bottom quintile of PC1 scores had significantly worse RFS, DSS, and OS. This cutoff may prove useful in identification of patients who are at risk in future studies.

It is plausible that PC1 will retain prognostic utility in other HNC cohorts. PC1 is derived from the relationship between recovery scores of HRQOL domains within a general HNC population who underwent definitive or adjuvant radiation therapy and that relationship should be reasonably consistent across similar populations. Using HRQOL recovery scores, independent groups could use PC1 as described here to assess its impact on survival within institutional cohorts. Ultimately, whether PC1 or de novo PCA would be necessary for other cohorts and/or disease sites remains unknown.

Interestingly, multivariate linear regression found N3 nodal stage and unplanned hospitalization to be correlated with PC1. Those with N3 disease were more likely to have higher PC1 scores and while this appears paradoxical from a prognostic standpoint, it is possible that relief of symptoms from bulky nodal disease following treatment corresponded with improved HRQOL metrics. Unplanned hospitalization, which previously has been shown to impact survival, was associated with lower PC1 scores [28]. Unplanned hospitalization is an attractive factor to identify at-risk patients who potentially may benefit from additional interventions. Notably, PC1 was not significantly correlated with KPS in this study. While prior reports have found HRQOL measures to correlate with performance status, typically, this corresponds to baseline scores [34]. As PC1 reflects the relationship between post-treatment and baseline HRQOL scores, these data would suggest that baseline performance status did not influence HRQOL recovery.

Future studies will focus on the prospective validation of PC1 as a prognostic factor in HNC, as well as the extent to which this effect can be modified with rehabilitation-based interventions such as physical/occupational therapy or counseling.

There are several limitations to our study. As this study required baseline and post-treatment HRQOL measures, many patients from prior analyses were excluded. As such, the study population is relatively smaller and the follow-up is limited, therefore, many variables found to influence the outcome of HNC patients in prior reports were not significant in the current investigation [28,29,35]. This can be a common challenge when investigating HRQOL measures. Furthermore, as PC1 requires three months post treatment to be determined, this would delay any rehabilitation-based interventions, potentially limiting its utility. Moreover, while the proportion of HNC subsites is consistent with an academic practice within the United States, our results may not be generalizable to other locations, particularly within Asia, where the incidence of nasopharyngeal cancer is far higher. Lastly, our findings may not be applicable to other HRQOL measures, such as the Short Form-36.

## 5. Conclusions

Our study provides evidence that post-treatment recovery of HRQOL domains was associated with DSS and OS in HNC. PC1 is an attractive clinical tool to assess the recovery across multiple different HRQOL and the relationship with survival. Prospective validation of the clinical utility of PC1 as a prognostic tool is warranted.

## Figures and Tables

**Table 1:** Patient demographics.

		All Patients (*n* = 218)		
		Median	*n*	%
Age		61.40		
Gender	Male		173	79.4%
	Female		45	20.6%
KPS	60		2	0.9%
	70		11	5.0%
	80		41	18.8%
	90		98	45.0%
	100		66	30.3%
Smoking status	Never		61	28.0%
	Former		143	65.6%
	Current		14	6.4%
Primary	Oral Cavity		16	7.3%
	Pharynx		126	57.8%
	Larynx		48	22.0%
	Salivary Glands		5	2.3%
	Unknown 1^0^		19	8.7%
	Other		4	1.8%
T stage	T0		18	8.3%
	T1		34	15.7%
	T2		63	29.0%
	T3		65	30.0%
	T4		37	17.1%
N stage	N0		42	19.4%
	N1		27	12.5%
	N2		126	58.3%
	N3		21	9.7%
M stage	M0		216	99.1%
	M1		2	0.9%
Histologic grade (differentiation)	Well		12	5.5%
	Moderate		64	29.4%
	Poorly		66	30.3%
	Unknown		76	34.9%
HPV status	Negative		42	19.4%
	Positive		108	49.8%
	Unknown		68	31.2%
Induction chemotherapy	No		206	94.5%
	Yes		12	5.5%
Concurrent chemotherapy	No		22	10.1%
	Yes		196	89.9%
Prior surgery	No		167	76.6%
	Yes		51	23.4%
Nutritional support	No		111	50.9%
	Yes		107	49.1%
Hospitalized	No		165	75.6%
	Yes		53	24.3%
Relapse	No		170	78.0%
	Yes		48	22.0%
Vital status	Alive		176	80.7%
	Dead		42	19.3%
Follow up (months)		24.78		

Karnofsky performance status (KPS); Primary (1^0^); Human papilloma virus (HPV).

**Table 2:** Baseline, post-treatment, and recovery health-related quality of life (HRQOL) metrics.

	Pre-Treatment (Median, IQR)	Post-Treatment (Median, IQR)	*p*-Value	Recovery (Complete, More Than 80%, <80%)	Recovery Mean (Std. Dev.)
**Functional Domains**					
Physical	93.3 (80–100)	86.7 (73.3–100)	0.0001	114 (52.3%), 66 (30.3%), 38 (17.4%)	0.651 (0.760)
Role	100 (66.7–100)	83.3 (66.7–100)	0.001	134 (61.5%), 24 (11.1%), 60 (27.5%)	0.660 (0.882)
Social	100 (66.7–100)	83.3 (66.7–100)	0.34	152 (69.7%), 21 (9.6%), 45 (20.6%)	0.509 (0.816)
Cognitive	100 (83.3–100)	100 (83.3–100)	0.99	169 (77.5%), 31 (14.2%), 18 (8.3%)	0.307 (0.616)
Emotional	83.3 (66.7–91.7)	83.3 (66.7–91.7)	0.34	149 (68.3%), 34 (15.6%), 35 (16.1%)	0.477 (0.757)
**Global Health Status**	75 (58.3–83.3)	70.8 (58.3–83.3)	0.1	121 (55.5%), 53 (24.3%), 43 (19.7%)	0.647 (0.797)

Interquartile range (IQR); Standard deviation (Std. Dev.).

**Table 3:** Univariate Cox regression.

	Univariate								
	RFS			DSS			OS		
	HR	95% CI	*p*-Value	HR	95% CI	*p*-Value	HR	95% CI	*p*-Value
Age	1	0.97–1.03	0.91	1.01	0.97–1.1	0.616	1.024	0.99–1.0	0.157
Gender	1.08	0.54–2.2	0.836	0.57	0.17–1.9	0.368	1	0.46–2.2	0.992
KPS	0.84	0.61–1.2	0.288	0.78	0.51–1.2	0.240	0.66	0.49–0.89	0.006
Primary									
Oral cavity	ref			ref			ref		
Pharynx	0.43	0.18–1.0	0.061	0.24	0.08–0.75	0.014	0.289	0.08–0.72	0.008
Larynx	0.58	0.22–1.5	0.274	0.69	0.22–2.2	0.534	0.645	0.25–1.7	0.371
Salivary glands	0	0-inf	0.966	0	0-inf	0.978	0.186	0.04–0.93	0.040
Unknown 1^0^	0.47	0.06–3.9	0.484	0	0-inf	0.990	0	0-inf	0.978
Other	0	0-inf	0.989	0	0-inf	0.994	0	0-inf	0.986
T stage									
T0	0	0-inf	0.967	0	0-inf	0.977	0.19	0.04–0.82	0.026
T1	0.43	0.13–1.4	0.156	0.1	0.01–0.82	0.032	0.17	0.05–0.58	0.005
T2	0.88	0.39–2.0	0.763	0.34	0.12–0.98	0.046	0.25	0.11–0.59	0.001
T3	1.13	0.51–2.5	0.763	0.51	0.20–1.3	0.161	0.4	0.19–0.83	0.014
T4	ref			ref			ref		
N stage									
N0	ref						ref		
N1	0.5	0.26–2.8	0.792	1.2	0.35–4.4	0.750	1.2	0.42–3.3	0.764
N2	1.17	0.53–2.6	0.705	0.72	0.27–1.9	0.495	0.78	0.36–1.7	0.514
N3	2.55	0.95–6.8	0.062	0.37	0.04–3.1	0.359	0.96	0.29–3.1	0.940
M stage	0.05	0-inf	0.674	0.05	0-inf	0.948	0.05	0-inf	0.926
Grade									
Well	0	0-inf	0.966	0	0-inf	0.177	0.78	0.10–6.1	0.814
Moderate	1.36	0.69–2.7	0.379	1.9	0.73–5.0	0.190	1.72	0.78–3.8	0.180
Poorly	1.3	0.55–2.2	0.775	1.3	0.50–3.7	0.583	1.72	0.80–3.7	0.160
Unknown	ref			ref			ref		
HPV status									
Negative	1.1	0.55–2.4	0.724	0.8	0.29–2.2	0.651	0.97	0.47–2.0	0.930
Positive	0.6	0.31–1.2	0.124	0.37	0.15–0.92	0.032	0.34	0.16–0.70	0.004
Unknown	ref			ref			ref		
Induction chemo	0.88	0.21–3.6	0.854	0.87	0.12–6.5	0.893	2.2	0.77–6.1	0.140
Concurrent chemo	1.2	0.37–3.9	0.758	1.7	0.22–12.4	0.617	0.68	0.24–1.9	0.470
Smoking									
Never	0.32	0.12–0.88	0.28	0.15	0.03–0.66	0.013	0.26	0.09–0.76	0.014
Former	0.48	0.20–1.1	0.097	0.42	0.14–1.3	0.125	0.43	0.18–1.1	0.064
Current	ref						ref		
Anticoagulant use	0.45	0.11–1.9	0.212	0.04	0.2–21.5	0.321	1.44	0.56–3.7	0.447
NSAID use	1.08	0.61–1.9	0.79	1	0.47–2.3	0.945	0.96	0.52–1.8	0.899
Prior surgery	1.25	0.64–2.5	0.517	1.4	0.57–3.6	0.450	1.2	0.57–2.5	0.620
Nutritional support	1.7	0.93–2.9	0.09	2.6	1.1–6.0	0.028	2.6	1.4–5.1	0.004
Hospitalized	1.2	0.66–2.3	0.508	1.4	0.60–3.2	0.452	1.6	0.83–3.2	0.141
Financial toxicity	1.2	0.56–2.6	0.632	1.6	0.59–4.2	0.365	1.5	0.69–3.2	0.308
Recovery									
PF	0.61	0.76–1.6	0.608	1.3	0.78–2.2	0.310	1.3	0.91–2.0	0.142
RF	1.3	0.96–1.8	0.095	1.4	0.91–2.1	0.128	1.6	1.1–2.2	0.009
SF	0.73	0.48–1.1	0.135	0.61	0.31–1.2	0.147	1	0.69–1.5	0.956
CF	1.4	0.94–2.1	0.097	1.4	0.8–2.6	0.225	1.4	0.92–2.2	0.115
EF	1.3	0.89–1.8	0.191	1.6	0.98–2.5	0.063	1.8	1.3–2.6	0.001
GQOL	1.3	0.92–1.8	0.149	1.8	1.1–2.9	0.015	1.7	1.2–2.4	0.006
PC1	0.83	0.70–0.99	0.039	0.74	0.59–0.94	0.013	0.74	0.62–0.89	0.001

Karnofsky performance status (KPS); Primary (1^0^); Human papilloma virus (HPV); Non-steroidal anti-inflammatory drugs (NSAID); Physical functioning (PF); Role functioning (RF); Social functioning (SF); Cognitive functioning (CF); Emotional functioning (EF), Global health status (GQOL); Principal component (PC).

**Table 4:** Multivariate Cox regression.

	Multivariate								
	RFS			DSS			OS		
	HR	95% CI	*p*-Value	HR	95% CI	*p*-Value	HR	95% CI	*p*-Value
KPS							0.76	0.53–1.1	0.148
Primary									
Oral cavity	ref			ref			ref		
Pharynx	0.48	0.17–1.3	0.153	0.24	0.08–0.75	0.014	0.58	0.19–1.7	0.325
Larynx	0.76	0.26–2.3	0.624	0.69	0.22–2.2	0.534	0.61	0.20–1.9	0.381
Salivary glands	0	0-inf	0.969	0	0-inf	0.978	0.34	0.06–2.1	0.239
Unknown 1^0^	0.69	0.08–6.3	0.74	0	0-inf	0.990	0	0-inf	0.979
Other	0	0-inf	0.993	0	0-inf	0.994	0	0-inf	0.989
T stage									
T0				*	*	*	*	*	*
T1				0.184	0.02–1.6	0.125	0.27	0.07–0.99	0.049
T2				0.54	0.17–1.7	0.297	0.43	0.17–1.1	0.080
T3				0.681	0.25–1.9	0.452	0.61	0.28–1.4	0.228
T4				ref			ref		
N stage									
N0	ref								
N1	1.7	0.48–6.1	0.407						
N2	1.8	0.75–4.4	0.187						
N3	4.1	1.4–11.8	0.010						
HPV status									
Negative				0.89	0.30–2.6	0.825	1	0.47–2.3	0.348
Positive				0.85	0.24–3.0	0.798	0.58	0.19–1.5	0.224
Unknown				ref			ref		
Smoking									
Never	0.42	0.14–1.2	0.112	0.34	0.67–1.8	0.199	0.57	0.18–1.8	0.348
Former	0.56	0.21–1.5	0.24	0.6	0.16–2.2	0.434	0.53	0.19–1.5	0.224
Current	ref			ref			ref		
Nutritional support	1.1	0.62–2.1	0.67	1.5	0.60–3.6	0.396	1.7	0.83–3.5	0.144
PC1	0.84	0.69–1.0	0.068	0.77	0.61–0.98	0.036	0.76	0.63–0.91	0.004

* T0 and Unknown 1^0^ are linear covariates. Karnofsky performance status (KPS); Primary (1^0^); Human papilloma virus (HPV); Principal component (PC); Confidence interval (CI).

**Table 5:** Factors associated with PC1 by linear regression.

	Univariate			Multivariate		
	Estimate	Std. Error	*p*-Value	Estimate	Std. Error	*p*-Value
Age	−0.017	0.011	0.117			
Gender	−0.106	0.259	0.684			
KPS	0.068	0.119	0.569			
Primary						
Oral cavity	ref			ref		
Pharynx	0.361	0.41	0.378	0.335	0.401	0.404
Larynx	0.176	0.446	0.693	0.134	0.432	0.755
Salivary glands	1.012	0.524	0.545	0.819	1.521	0.591
Unknown 1^0^	−0.217	0.791	0.784	−0.564	0.764	0.461
Other	0.445	0.863	0.607	−1.34	1.14	0.241
T stage						
T0	ref			ref		
T1	−0.722	0.439	0.1	0.194	1.54	0.899
T2	−0.918	0.401	0.023	0.079	1.51	0.958
T3	−0.557	0.399	0.165	0.648	1.53	0.672
T4	−1.063	0.433	0.015	0.303	1.54	0.844
N stage						
N0	ref			ref		
N1	0.406	0.377	0.283	0.504	0.39	0.198
N2	0.103	0.27	0.704	0.378	0.302	0.212
N3	0.718	0.409	0.081	1.07	0.429	0.013
M stage	−1.344	1.097	0.222			
HPV status						
Negative	ref					
Positive	0.223	0.282	0.43			
Unknown	0.225	0.304	0.46			
Induction chemo	−0.352	0.46	0.444			
Concurrent chemo	−0.34	0.348	0.329			
Smoking						
Never	ref					
Former	−0.268	0.235	0.257			
Current	0.219	0.457	0.632			
Anticoagulant use	0.228	0.363	0.531			
NSAID use	−0.372	0.208	0.076	−0.354	0.2	0.083
Prior surgery	0.073	0.086	0.392			
Nutritional support	−0.578	0.206	0.006	−0.38	0.218	0.079
Hospitalized	−0.997	0.235	<0.001	−0.997	0.244	<0.001
Financial toxicity	−0.008	0.305	0.718			

Karnofsky performance status (KPS); Primary (1^0^); Human papilloma virus (HPV); Non-steroidal anti-inflammatory drugs (NSAID); Chemotherapy (chemo); Standard (Std.)

## Data Availability

Farrugia and Singh had full access to all the data in the study and take responsibility for the integrity of the data and the accuracy of the data analysis. The data underlying this article cannot be shared publicly for the privacy of individuals that participated in the study. The data are available from the corresponding author upon reasonable request.

## Supplementary Materials

The following are available online at https://www.mdpi.com/2072-6694/13/5/1155/s1, Table S1: Loading matrix, Document S1: EORTC-QLQ-C30.
